# Supplementary material for: Would Climate Change Influence the Potential Distribution and Ecological Niche of Bluetongue Virus and Its Main Vector in Peru?
Source: Viruses. 2023 Mar 30;15(4):892. doi: 10.3390/v15040892 (PMC10145190; doi:10.3390/v15040892)
Supplement: Supplementary file 1 [file viruses-15-00892-s001.zip › viruses-2297424-supplementary.pdf]

Table S1. Occurrence records of bluetongue virus and *Culicoides insignis*.

Species	Longitude	Latitude	Source
Bluetongue virus	-75.709932	-11.769377	Sample was collected by Servicio Nacional de Sanidad Agraria del Perú (SENASA) and was analyzed by Virology laboratory at Universidad Nacional Mayor de San Marcos (UNMSM)
Bluetongue virus	-74.459268	-12.862998	Sample was collected by Servicio Nacional de Sanidad Agraria del Perú (SENASA) and was analyzed by Virology laboratory at Universidad Nacional Mayor de San Marcos (UNMSM)
Bluetongue virus	-74.35912	-13.83055	Sample was collected by Servicio Nacional de Sanidad Agraria del Perú (SENASA) and was analyzed by Virology laboratory at Universidad Nacional Mayor de San Marcos (UNMSM)
Bluetongue virus	-71.560352	-13.482446	Sample was collected by Servicio Nacional de Sanidad Agraria del Perú (SENASA) and was analyzed by Virology laboratory at Universidad Nacional Mayor de San Marcos (UNMSM)
Bluetongue virus	-72.205376	-13.414435	Sample was collected by Servicio Nacional de Sanidad Agraria del Perú (SENASA) and was analyzed by Virology laboratory at Universidad Nacional Mayor de San Marcos (UNMSM)
Bluetongue virus	-76.760009	-9.382051	Sample was collected by Servicio Nacional de Sanidad Agraria del Perú (SENASA) and was analyzed by Virology laboratory at Universidad Nacional Mayor de San Marcos (UNMSM)
Bluetongue virus	-74.23429	-14.61996	Sample was collected by Servicio Nacional de Sanidad Agraria del Perú (SENASA) and was analyzed by Virology laboratory at Universidad Nacional Mayor de San Marcos (UNMSM)
Bluetongue virus	-75.304166	-11.939722	Sample was collected by Servicio Nacional de Sanidad Agraria del Perú (SENASA) and was analyzed by Virology laboratory at Universidad Nacional Mayor de San Marcos (UNMSM)
Bluetongue virus	-72.085005	-14.403343	Sample was collected by Servicio Nacional de Sanidad Agraria del Perú (SENASA) and was analyzed by Virology laboratory at Universidad Nacional Mayor de San Marcos (UNMSM)
Bluetongue virus	-78.421284	-7.154782	Sample was collected by Servicio Nacional de Sanidad Agraria del Perú (SENASA) and was analyzed by Virology laboratory at Universidad Nacional Mayor de San Marcos (UNMSM)
Bluetongue virus	-74.002966	-13.618381	Sample was collected by Servicio Nacional de Sanidad Agraria del Perú (SENASA) and was analyzed by Virology laboratory at Universidad Nacional Mayor de San Marcos (UNMSM)
Bluetongue virus	-74.99405	-12.502295	Sample was collected by Servicio Nacional de Sanidad Agraria del Perú (SENASA) and was analyzed by Virology laboratory at Universidad Nacional Mayor de San Marcos (UNMSM)
Bluetongue virus	-74.54503	-12.517876	Sample was collected by Servicio Nacional de Sanidad Agraria del Perú (SENASA) and was analyzed by Virology laboratory at Universidad Nacional Mayor de San Marcos (UNMSM)
Bluetongue virus	-78.072	-7.032278	Sample was collected by Servicio Nacional de Sanidad Agraria del Perú (SENASA) and was analyzed by Virology laboratory at Universidad Nacional Mayor de San Marcos (UNMSM)
Bluetongue virus	-78.539	-6.657	Sample was collected by Servicio Nacional de Sanidad Agraria del Perú (SENASA) and was analyzed by Virology laboratory at Universidad Nacional Mayor de San Marcos (UNMSM)
Bluetongue virus	-79.5252	-5.0306	Sample was collected by Servicio Nacional de Sanidad Agraria del Perú (SENASA) and was analyzed by Virology laboratory at Universidad Nacional Mayor de San Marcos (UNMSM)
Bluetongue virus	-79.064829	-6.515411	Sample was collected by Servicio Nacional de Sanidad Agraria del Perú (SENASA) and was analyzed by Virology laboratory at Universidad Nacional Mayor de San Marcos (UNMSM)
Bluetongue virus	-77.865687	-6.176727	Sample was collected by Servicio Nacional de Sanidad Agraria del Perú (SENASA) and was analyzed by Virology laboratory at Universidad Nacional Mayor de San Marcos (UNMSM)

Bluetongue virus	-78.915401	-6.643821	Sample was collected by Servicio Nacional de Sanidad Agraria del Perú (SENASA) and was analyzed by Virology laboratory at Universidad Nacional Mayor de San Marcos (UNMSM)
Bluetongue virus	-70.971826	-16.674883	Sample was collected by Servicio Nacional de Sanidad Agraria del Perú (SENASA) and was analyzed by Virology laboratory at Universidad Nacional Mayor de San Marcos (UNMSM)
Bluetongue virus	-78.51337	-5.88027	Sample was collected by Servicio Nacional de Sanidad Agraria del Perú (SENASA) and was analyzed by Virology laboratory at Universidad Nacional Mayor de San Marcos (UNMSM)
Bluetongue virus	-78.597819	-6.3877	Sample was collected by Servicio Nacional de Sanidad Agraria del Perú (SENASA) and was analyzed by Virology laboratory at Universidad Nacional Mayor de San Marcos (UNMSM)
Bluetongue virus	-79.460278	-5.940556	Sample was collected by Servicio Nacional de Sanidad Agraria del Perú (SENASA) and was analyzed by Virology laboratory at Universidad Nacional Mayor de San Marcos (UNMSM)
Bluetongue virus	-79.61861111	-4.6738889	Sample was collected by Servicio Nacional de Sanidad Agraria del Perú (SENASA) and was analyzed by Virology laboratory at Universidad Nacional Mayor de San Marcos (UNMSM)
Bluetongue virus	-78.78091	-6.00983	Sample was collected by Servicio Nacional de Sanidad Agraria del Perú (SENASA) and was analyzed by Virology laboratory at Universidad Nacional Mayor de San Marcos (UNMSM)
Bluetongue virus	-75.444277	-10.61067	Sample was collected by Servicio Nacional de Sanidad Agraria del Perú (SENASA) and was analyzed by Virology laboratory at Universidad Nacional Mayor de San Marcos (UNMSM)
Bluetongue virus	-78.676221	-6.159251	Sample was collected by Servicio Nacional de Sanidad Agraria del Perú (SENASA) and was analyzed by Virology laboratory at Universidad Nacional Mayor de San Marcos (UNMSM)
Bluetongue virus	-79.432561	-5.29532	Sample was collected by Servicio Nacional de Sanidad Agraria del Perú (SENASA) and was analyzed by Virology laboratory at Universidad Nacional Mayor de San Marcos (UNMSM)
Bluetongue virus	-78.85831	-6.246879	Sample was collected by Servicio Nacional de Sanidad Agraria del Perú (SENASA) and was analyzed by Virology laboratory at Universidad Nacional Mayor de San Marcos (UNMSM)
Bluetongue virus	-79.944673	-4.936012	Sample was collected by Servicio Nacional de Sanidad Agraria del Perú (SENASA) and was analyzed by Virology laboratory at Universidad Nacional Mayor de San Marcos (UNMSM)
Bluetongue virus	-75.341227	-10.8887276	Sample was collected by Servicio Nacional de Sanidad Agraria del Perú (SENASA) and was analyzed by Virology laboratory at Universidad Nacional Mayor de San Marcos (UNMSM)
Bluetongue virus	-75.870498	-9.464807	Sample was collected by Servicio Nacional de Sanidad Agraria del Perú (SENASA) and was analyzed by Virology laboratory at Universidad Nacional Mayor de San Marcos (UNMSM)
Bluetongue virus	-79.546448	-5.49857	Sample was collected by Servicio Nacional de Sanidad Agraria del Perú (SENASA) and was analyzed by Virology laboratory at Universidad Nacional Mayor de San Marcos (UNMSM)
Bluetongue virus	-75.17238	-10.42681	Sample was collected by Servicio Nacional de Sanidad Agraria del Perú (SENASA) and was analyzed by Virology laboratory at Universidad Nacional Mayor de San Marcos (UNMSM)
Bluetongue virus	-78.820618	-5.138789	Sample was collected by Servicio Nacional de Sanidad Agraria del Perú (SENASA) and was analyzed by Virology laboratory at Universidad Nacional Mayor de San Marcos (UNMSM)
Bluetongue virus	-73.504326	-12.906735	Sample was collected by Servicio Nacional de Sanidad Agraria del Perú (SENASA) and was analyzed by Virology laboratory at Universidad Nacional Mayor de San Marcos (UNMSM)
Bluetongue virus	-79.825672	-4.62981	Sample was collected by Servicio Nacional de Sanidad Agraria del Perú (SENASA) and was analyzed by Virology laboratory at Universidad Nacional Mayor de San Marcos (UNMSM)

Bluetongue virus	-72.685135	-12.806757	Sample was collected by Servicio Nacional de Sanidad Agraria del Perú (SENASA) and was analyzed by Virology laboratory at Universidad Nacional Mayor de San Marcos (UNMSM)
Bluetongue virus	-72.693062	-11.903184	Sample was collected by Servicio Nacional de Sanidad Agraria del Perú (SENASA) and was analyzed by Virology laboratory at Universidad Nacional Mayor de San Marcos (UNMSM)
Bluetongue virus	-78.76633	-7.21668	Sample was collected by Servicio Nacional de Sanidad Agraria del Perú (SENASA) and was analyzed by Virology laboratory at Universidad Nacional Mayor de San Marcos (UNMSM)
Bluetongue virus	-79.231019	-5.931002	Sample was collected by Servicio Nacional de Sanidad Agraria del Perú (SENASA) and was analyzed by Virology laboratory at Universidad Nacional Mayor de San Marcos (UNMSM)
Bluetongue virus	-74.21174	-10.743445	Sample was collected by Servicio Nacional de Sanidad Agraria del Perú (SENASA) and was analyzed by Virology laboratory at Universidad Nacional Mayor de San Marcos (UNMSM)
Bluetongue virus	-79.011918	-5.839939	Sample was collected by Servicio Nacional de Sanidad Agraria del Perú (SENASA) and was analyzed by Virology laboratory at Universidad Nacional Mayor de San Marcos (UNMSM)
Bluetongue virus	-74.323591	-11.238305	Sample was collected by Servicio Nacional de Sanidad Agraria del Perú (SENASA) and was analyzed by Virology laboratory at Universidad Nacional Mayor de San Marcos (UNMSM)
Bluetongue virus	-79.452474	-6.318295	Sample was collected by Servicio Nacional de Sanidad Agraria del Perú (SENASA) and was analyzed by Virology laboratory at Universidad Nacional Mayor de San Marcos (UNMSM)
Bluetongue virus	-75.998436	-6.889504	Sample was collected by Servicio Nacional de Sanidad Agraria del Perú (SENASA) and was analyzed by Virology laboratory at Universidad Nacional Mayor de San Marcos (UNMSM)
Bluetongue virus	-75.335222	-11.198374	Sample was collected by Servicio Nacional de Sanidad Agraria del Perú (SENASA) and was analyzed by Virology laboratory at Universidad Nacional Mayor de San Marcos (UNMSM)
Bluetongue virus	-76.411942	-9.27791	Sample was collected by Servicio Nacional de Sanidad Agraria del Perú (SENASA) and was analyzed by Virology laboratory at Universidad Nacional Mayor de San Marcos (UNMSM)
Bluetongue virus	-76.758294	-6.285838	Sample was collected by Servicio Nacional de Sanidad Agraria del Perú (SENASA) and was analyzed by Virology laboratory at Universidad Nacional Mayor de San Marcos (UNMSM)
Bluetongue virus	-78.624643	-5.451458	Sample was collected by Servicio Nacional de Sanidad Agraria del Perú (SENASA) and was analyzed by Virology laboratory at Universidad Nacional Mayor de San Marcos (UNMSM)
Bluetongue virus	-79.05553	-7.306223	Sample was collected by Servicio Nacional de Sanidad Agraria del Perú (SENASA) and was analyzed by Virology laboratory at Universidad Nacional Mayor de San Marcos (UNMSM)
Bluetongue virus	-77.473141	-5.790595	Sample was collected by Servicio Nacional de Sanidad Agraria del Perú (SENASA) and was analyzed by Virology laboratory at Universidad Nacional Mayor de San Marcos (UNMSM)
Bluetongue virus	-75.123178	-10.665298	Sample was collected by Servicio Nacional de Sanidad Agraria del Perú (SENASA) and was analyzed by Virology laboratory at Universidad Nacional Mayor de San Marcos (UNMSM)
Bluetongue virus	-77.04825	-5.77204	Sample was collected by Servicio Nacional de Sanidad Agraria del Perú (SENASA) and was analyzed by Virology laboratory at Universidad Nacional Mayor de San Marcos (UNMSM)
Bluetongue virus	-78.43453	-5.61005	Sample was collected by Servicio Nacional de Sanidad Agraria del Perú (SENASA) and was analyzed by Virology laboratory at Universidad Nacional Mayor de San Marcos (UNMSM)
Bluetongue virus	-77.085297	-6.044791	Sample was collected by Servicio Nacional de Sanidad Agraria del Perú (SENASA) and was analyzed by Virology laboratory at Universidad Nacional Mayor de San Marcos (UNMSM)

Bluetongue virus	-77.267678	-6.004355	Sample was collected by Servicio Nacional de Sanidad Agraria del Perú (SENASA) and was analyzed by Virology laboratory at Universidad Nacional Mayor de San Marcos (UNMSM)
Bluetongue virus	-75.540027	-10.043469	Sample was collected by Servicio Nacional de Sanidad Agraria del Perú (SENASA) and was analyzed by Virology laboratory at Universidad Nacional Mayor de San Marcos (UNMSM)
Bluetongue virus	-76.177945	-9.09272	Sample was collected by Servicio Nacional de Sanidad Agraria del Perú (SENASA) and was analyzed by Virology laboratory at Universidad Nacional Mayor de San Marcos (UNMSM)
Bluetongue virus	-78.823843	-5.471803	Sample was collected by Servicio Nacional de Sanidad Agraria del Perú (SENASA) and was analyzed by Virology laboratory at Universidad Nacional Mayor de San Marcos (UNMSM)
Bluetongue virus	-77.774833	-3.369486	Sample was collected by Servicio Nacional de Sanidad Agraria del Perú (SENASA) and was analyzed by Virology laboratory at Universidad Nacional Mayor de San Marcos (UNMSM)
Bluetongue virus	-75.989271	-9.235127	Sample was collected by Servicio Nacional de Sanidad Agraria del Perú (SENASA) and was analyzed by Virology laboratory at Universidad Nacional Mayor de San Marcos (UNMSM)
Bluetongue virus	-74.697062	-11.016566	Sample was collected by Servicio Nacional de Sanidad Agraria del Perú (SENASA) and was analyzed by Virology laboratory at Universidad Nacional Mayor de San Marcos (UNMSM)
Bluetongue virus	-76.479975	-6.562686	Sample was collected by Servicio Nacional de Sanidad Agraria del Perú (SENASA) and was analyzed by Virology laboratory at Universidad Nacional Mayor de San Marcos (UNMSM)
Bluetongue virus	-76.451878	-8.455485	Sample was collected by Servicio Nacional de Sanidad Agraria del Perú (SENASA) and was analyzed by Virology laboratory at Universidad Nacional Mayor de San Marcos (UNMSM)
Bluetongue virus	-73.850015	-12.520118	Sample was collected by Servicio Nacional de Sanidad Agraria del Perú (SENASA) and was analyzed by Virology laboratory at Universidad Nacional Mayor de San Marcos (UNMSM)
Bluetongue virus	-76.345429	-8.305323	Sample was collected by Servicio Nacional de Sanidad Agraria del Perú (SENASA) and was analyzed by Virology laboratory at Universidad Nacional Mayor de San Marcos (UNMSM)
Bluetongue virus	-71.360647	-12.861532	Sample was collected by Servicio Nacional de Sanidad Agraria del Perú (SENASA) and was analyzed by Virology laboratory at Universidad Nacional Mayor de San Marcos (UNMSM)
Bluetongue virus	-75.356178	-10.069149	Sample was collected by Servicio Nacional de Sanidad Agraria del Perú (SENASA) and was analyzed by Virology laboratory at Universidad Nacional Mayor de San Marcos (UNMSM)
Bluetongue virus	-79.925476	-4.422246	Sample was collected by Servicio Nacional de Sanidad Agraria del Perú (SENASA) and was analyzed by Virology laboratory at Universidad Nacional Mayor de San Marcos (UNMSM)
Bluetongue virus	-76.733197	-6.699466	Sample was collected by Servicio Nacional de Sanidad Agraria del Perú (SENASA) and was analyzed by Virology laboratory at Universidad Nacional Mayor de San Marcos (UNMSM)
Bluetongue virus	-70.52716	-12.994241	Sample was collected by Servicio Nacional de Sanidad Agraria del Perú (SENASA) and was analyzed by Virology laboratory at Universidad Nacional Mayor de San Marcos (UNMSM)
Bluetongue virus	-78.39837	-5.2027	Sample was collected by Servicio Nacional de Sanidad Agraria del Perú (SENASA) and was analyzed by Virology laboratory at Universidad Nacional Mayor de San Marcos (UNMSM)
Bluetongue virus	-75.724974	-14.081825	Sample was collected by Servicio Nacional de Sanidad Agraria del Perú (SENASA) and was analyzed by Virology laboratory at Universidad Nacional Mayor de San Marcos (UNMSM)
Bluetongue virus	-76.668508	-7.48274	Sample was collected by Servicio Nacional de Sanidad Agraria del Perú (SENASA) and was analyzed by Virology laboratory at Universidad Nacional Mayor de San Marcos (UNMSM)

Bluetongue virus	-79.278329	-6.613676	Sample was collected by Servicio Nacional de Sanidad Agraria del Perú (SENASA) and was analyzed by Virology laboratory at Universidad Nacional Mayor de San Marcos (UNMSM)
Bluetongue virus	-80.112162	-4.412691	Sample was collected by Servicio Nacional de Sanidad Agraria del Perú (SENASA) and was analyzed by Virology laboratory at Universidad Nacional Mayor de San Marcos (UNMSM)
Bluetongue virus	-76.803986	-6.868973	Sample was collected by Servicio Nacional de Sanidad Agraria del Perú (SENASA) and was analyzed by Virology laboratory at Universidad Nacional Mayor de San Marcos (UNMSM)
Bluetongue virus	-75.454015	-9.076141	Sample was collected by Servicio Nacional de Sanidad Agraria del Perú (SENASA) and was analyzed by Virology laboratory at Universidad Nacional Mayor de San Marcos (UNMSM)
Bluetongue virus	-75.431947	-9.716688	Sample was collected by Servicio Nacional de Sanidad Agraria del Perú (SENASA) and was analyzed by Virology laboratory at Universidad Nacional Mayor de San Marcos (UNMSM)
Bluetongue virus	-75.175436	-10.239389	Sample was collected by Servicio Nacional de Sanidad Agraria del Perú (SENASA) and was analyzed by Virology laboratory at Universidad Nacional Mayor de San Marcos (UNMSM)
Bluetongue virus	-76.583499	-7.045916	Sample was collected by Servicio Nacional de Sanidad Agraria del Perú (SENASA) and was analyzed by Virology laboratory at Universidad Nacional Mayor de San Marcos (UNMSM)
Bluetongue virus	-69.49097	-11.357775	Sample was collected by Servicio Nacional de Sanidad Agraria del Perú (SENASA) and was analyzed by Virology laboratory at Universidad Nacional Mayor de San Marcos (UNMSM)
Bluetongue virus	-69.453881	-11.168131	Sample was collected by Servicio Nacional de Sanidad Agraria del Perú (SENASA) and was analyzed by Virology laboratory at Universidad Nacional Mayor de San Marcos (UNMSM)
Bluetongue virus	-69.547133	-11.010431	Sample was collected by Servicio Nacional de Sanidad Agraria del Perú (SENASA) and was analyzed by Virology laboratory at Universidad Nacional Mayor de San Marcos (UNMSM)
Bluetongue virus	-79.92905	-5.144751	Sample was collected by Servicio Nacional de Sanidad Agraria del Perú (SENASA) and was analyzed by Virology laboratory at Universidad Nacional Mayor de San Marcos (UNMSM)
Bluetongue virus	-76.442273	-7.279674	Sample was collected by Servicio Nacional de Sanidad Agraria del Perú (SENASA) and was analyzed by Virology laboratory at Universidad Nacional Mayor de San Marcos (UNMSM)
Bluetongue virus	-76.352047	-6.74058	Sample was collected by Servicio Nacional de Sanidad Agraria del Perú (SENASA) and was analyzed by Virology laboratory at Universidad Nacional Mayor de San Marcos (UNMSM)
Bluetongue virus	-75.362679	-8.914666	Sample was collected by Servicio Nacional de Sanidad Agraria del Perú (SENASA) and was analyzed by Virology laboratory at Universidad Nacional Mayor de San Marcos (UNMSM)
Bluetongue virus	-76.577388	-6.792936	Sample was collected by Servicio Nacional de Sanidad Agraria del Perú (SENASA) and was analyzed by Virology laboratory at Universidad Nacional Mayor de San Marcos (UNMSM)
Bluetongue virus	-69.02451	-12.10889	Sample was collected by Servicio Nacional de Sanidad Agraria del Perú (SENASA) and was analyzed by Virology laboratory at Universidad Nacional Mayor de San Marcos (UNMSM)
Bluetongue virus	-69.32725	-11.44711	Sample was collected by Servicio Nacional de Sanidad Agraria del Perú (SENASA) and was analyzed by Virology laboratory at Universidad Nacional Mayor de San Marcos (UNMSM)
Bluetongue virus	-74.97637	-10.249568	Sample was collected by Servicio Nacional de Sanidad Agraria del Perú (SENASA) and was analyzed by Virology laboratory at Universidad Nacional Mayor de San Marcos (UNMSM)
Bluetongue virus	-69.14641	-12.25125	Sample was collected by Servicio Nacional de Sanidad Agraria del Perú (SENASA) and was analyzed by Virology laboratory at Universidad Nacional Mayor de San Marcos (UNMSM)

Bluetongue virus	-75.069454	-9.944239	Sample was collected by Servicio Nacional de Sanidad Agraria del Perú (SENASA) and was analyzed by Virology laboratory at Universidad Nacional Mayor de San Marcos (UNMSM)
Bluetongue virus	-75.149657	-8.503905	Sample was collected by Servicio Nacional de Sanidad Agraria del Perú (SENASA) and was analyzed by Virology laboratory at Universidad Nacional Mayor de San Marcos (UNMSM)
Bluetongue virus	-77.82842	-4.67234	Sample was collected by Servicio Nacional de Sanidad Agraria del Perú (SENASA) and was analyzed by Virology laboratory at Universidad Nacional Mayor de San Marcos (UNMSM)
Bluetongue virus	-78.02664	-4.892651	Sample was collected by Servicio Nacional de Sanidad Agraria del Perú (SENASA) and was analyzed by Virology laboratory at Universidad Nacional Mayor de San Marcos (UNMSM)
Bluetongue virus	-79.74648	-5.326894	Sample was collected by Servicio Nacional de Sanidad Agraria del Perú (SENASA) and was analyzed by Virology laboratory at Universidad Nacional Mayor de San Marcos (UNMSM)
Bluetongue virus	-75.110393	-8.242663	Sample was collected by Servicio Nacional de Sanidad Agraria del Perú (SENASA) and was analyzed by Virology laboratory at Universidad Nacional Mayor de San Marcos (UNMSM)
Bluetongue virus	-73.797532	-10.700933	Sample was collected by Servicio Nacional de Sanidad Agraria del Perú (SENASA) and was analyzed by Virology laboratory at Universidad Nacional Mayor de San Marcos (UNMSM)
Bluetongue virus	-69.10291	-12.71004	Sample was collected by Servicio Nacional de Sanidad Agraria del Perú (SENASA) and was analyzed by Virology laboratory at Universidad Nacional Mayor de San Marcos (UNMSM)
Bluetongue virus	-69.14053	-12.96649	Sample was collected by Servicio Nacional de Sanidad Agraria del Perú (SENASA) and was analyzed by Virology laboratory at Universidad Nacional Mayor de San Marcos (UNMSM)
Bluetongue virus	-69.346609	-12.728181	Sample was collected by Servicio Nacional de Sanidad Agraria del Perú (SENASA) and was analyzed by Virology laboratory at Universidad Nacional Mayor de San Marcos (UNMSM)
Bluetongue virus	-69.12981	-12.45441	Sample was collected by Servicio Nacional de Sanidad Agraria del Perú (SENASA) and was analyzed by Virology laboratory at Universidad Nacional Mayor de San Marcos (UNMSM)
Bluetongue virus	-75.005935	-8.716977	Sample was collected by Servicio Nacional de Sanidad Agraria del Perú (SENASA) and was analyzed by Virology laboratory at Universidad Nacional Mayor de San Marcos (UNMSM)
Bluetongue virus	-80.4443	-5.2852	Sample was collected by Servicio Nacional de Sanidad Agraria del Perú (SENASA) and was analyzed by Virology laboratory at Universidad Nacional Mayor de San Marcos (UNMSM)
Bluetongue virus	-79.305918	-6.87119	Sample was collected by Servicio Nacional de Sanidad Agraria del Perú (SENASA) and was analyzed by Virology laboratory at Universidad Nacional Mayor de San Marcos (UNMSM)
Bluetongue virus	-74.744413	-8.915747	Sample was collected by Servicio Nacional de Sanidad Agraria del Perú (SENASA) and was analyzed by Virology laboratory at Universidad Nacional Mayor de San Marcos (UNMSM)
Bluetongue virus	-74.850473	-8.458544	Sample was collected by Servicio Nacional de Sanidad Agraria del Perú (SENASA) and was analyzed by Virology laboratory at Universidad Nacional Mayor de San Marcos (UNMSM)
Bluetongue virus	-76.492191	-5.806946	Sample was collected by Servicio Nacional de Sanidad Agraria del Perú (SENASA) and was analyzed by Virology laboratory at Universidad Nacional Mayor de San Marcos (UNMSM)
Bluetongue virus	-74.797966	-8.711983	Sample was collected by Servicio Nacional de Sanidad Agraria del Perú (SENASA) and was analyzed by Virology laboratory at Universidad Nacional Mayor de San Marcos (UNMSM)
Bluetongue virus	-80.3717	-4.73129	Sample was collected by Servicio Nacional de Sanidad Agraria del Perú (SENASA) and was analyzed by Virology laboratory at Universidad Nacional Mayor de San Marcos (UNMSM)

Bluetongue virus	-79.747503	-5.97796	Sample was collected by Servicio Nacional de Sanidad Agraria del Perú (SENASA) and was analyzed by Virology laboratory at Universidad Nacional Mayor de San Marcos (UNMSM)
Bluetongue virus	-75.081894	-7.089515	Sample was collected by Servicio Nacional de Sanidad Agraria del Perú (SENASA) and was analyzed by Virology laboratory at Universidad Nacional Mayor de San Marcos (UNMSM)
Bluetongue virus	-75.176103	-6.893882	Sample was collected by Servicio Nacional de Sanidad Agraria del Perú (SENASA) and was analyzed by Virology laboratory at Universidad Nacional Mayor de San Marcos (UNMSM)
Bluetongue virus	-80.41653	-5.0267	Sample was collected by Servicio Nacional de Sanidad Agraria del Perú (SENASA) and was analyzed by Virology laboratory at Universidad Nacional Mayor de San Marcos (UNMSM)
Bluetongue virus	-74.770279	-8.227088	Sample was collected by Servicio Nacional de Sanidad Agraria del Perú (SENASA) and was analyzed by Virology laboratory at Universidad Nacional Mayor de San Marcos (UNMSM)
Bluetongue virus	-74.307503	-8.610643	Sample was collected by Servicio Nacional de Sanidad Agraria del Perú (SENASA) and was analyzed by Virology laboratory at Universidad Nacional Mayor de San Marcos (UNMSM)
Bluetongue virus	-76.541485	-4.78673	Sample was collected by Servicio Nacional de Sanidad Agraria del Perú (SENASA) and was analyzed by Virology laboratory at Universidad Nacional Mayor de San Marcos (UNMSM)
Bluetongue virus	-74.635007	-8.395437	Sample was collected by Servicio Nacional de Sanidad Agraria del Perú (SENASA) and was analyzed by Virology laboratory at Universidad Nacional Mayor de San Marcos (UNMSM)
Bluetongue virus	-73.26509	-3.639037	Sample was collected by Servicio Nacional de Sanidad Agraria del Perú (SENASA) and was analyzed by Virology laboratory at Universidad Nacional Mayor de San Marcos (UNMSM)
Bluetongue virus	-79.83229	-5.79964	Sample was collected by Servicio Nacional de Sanidad Agraria del Perú (SENASA) and was analyzed by Virology laboratory at Universidad Nacional Mayor de San Marcos (UNMSM)
Bluetongue virus	-73.448841	-4.032504	Sample was collected by Servicio Nacional de Sanidad Agraria del Perú (SENASA) and was analyzed by Virology laboratory at Universidad Nacional Mayor de San Marcos (UNMSM)
Bluetongue virus	-73.646819	-4.917715	Sample was collected by Servicio Nacional de Sanidad Agraria del Perú (SENASA) and was analyzed by Virology laboratory at Universidad Nacional Mayor de San Marcos (UNMSM)
Bluetongue virus	-72.661216	-2.460614	Sample was collected by Servicio Nacional de Sanidad Agraria del Perú (SENASA) and was analyzed by Virology laboratory at Universidad Nacional Mayor de San Marcos (UNMSM)
Bluetongue virus	-79.690193	-6.301737	Sample was collected by Servicio Nacional de Sanidad Agraria del Perú (SENASA) and was analyzed by Virology laboratory at Universidad Nacional Mayor de San Marcos (UNMSM)
Bluetongue virus	-70.54611111	-4.0211111	Sample was collected by Servicio Nacional de Sanidad Agraria del Perú (SENASA) and was analyzed by Virology laboratory at Universidad Nacional Mayor de San Marcos (UNMSM)
Bluetongue virus	-80.64228	-3.867303	Sample was collected by Servicio Nacional de Sanidad Agraria del Perú (SENASA) and was analyzed by Virology laboratory at Universidad Nacional Mayor de San Marcos (UNMSM)
Bluetongue virus	-80.136491	-5.167048	Sample was collected by Servicio Nacional de Sanidad Agraria del Perú (SENASA) and was analyzed by Virology laboratory at Universidad Nacional Mayor de San Marcos (UNMSM)
Bluetongue virus	-73.117844	-3.510528	Sample was collected by Servicio Nacional de Sanidad Agraria del Perú (SENASA) and was analyzed by Virology laboratory at Universidad Nacional Mayor de San Marcos (UNMSM)
Bluetongue virus	-73.3403	-3.809656	Sample was collected by Servicio Nacional de Sanidad Agraria del Perú (SENASA) and was analyzed by Virology laboratory at Universidad Nacional Mayor de San Marcos (UNMSM)

Bluetongue virus	-80.194594	-4.993851	Sample was collected by Servicio Nacional de Sanidad Agraria del Perú (SENASA) and was analyzed by Virology laboratory at Universidad Nacional Mayor de San Marcos (UNMSM)
Bluetongue virus	-77.577886	-11.108464	Sample was collected by Servicio Nacional de Sanidad Agraria del Perú (SENASA) and was analyzed by Virology laboratory at Universidad Nacional Mayor de San Marcos (UNMSM)
Bluetongue virus	-80.917973	-4.064093	Sample was collected by Servicio Nacional de Sanidad Agraria del Perú (SENASA) and was analyzed by Virology laboratory at Universidad Nacional Mayor de San Marcos (UNMSM)
Bluetongue virus	-80.670267	-4.988359	Sample was collected by Servicio Nacional de Sanidad Agraria del Perú (SENASA) and was analyzed by Virology laboratory at Universidad Nacional Mayor de San Marcos (UNMSM)
Bluetongue virus	-80.753224	-4.81419	Sample was collected by Servicio Nacional de Sanidad Agraria del Perú (SENASA) and was analyzed by Virology laboratory at Universidad Nacional Mayor de San Marcos (UNMSM)
Bluetongue virus	-79.841021	-6.47356	Sample was collected by Servicio Nacional de Sanidad Agraria del Perú (SENASA) and was analyzed by Virology laboratory at Universidad Nacional Mayor de San Marcos (UNMSM)
Bluetongue virus	-76.868082	-12.237933	Sample was collected by Servicio Nacional de Sanidad Agraria del Perú (SENASA) and was analyzed by Virology laboratory at Universidad Nacional Mayor de San Marcos (UNMSM)
Bluetongue virus	-80.653262	-3.672445	Sample was collected by Servicio Nacional de Sanidad Agraria del Perú (SENASA) and was analyzed by Virology laboratory at Universidad Nacional Mayor de San Marcos (UNMSM)
Bluetongue virus	-80.667459	-5.208379	Sample was collected by Servicio Nacional de Sanidad Agraria del Perú (SENASA) and was analyzed by Virology laboratory at Universidad Nacional Mayor de San Marcos (UNMSM)
Bluetongue virus	-80.926224	-4.922414	Sample was collected by Servicio Nacional de Sanidad Agraria del Perú (SENASA) and was analyzed by Virology laboratory at Universidad Nacional Mayor de San Marcos (UNMSM)
Bluetongue virus	-80.265469	-3.503695	Sample was collected by Servicio Nacional de Sanidad Agraria del Perú (SENASA) and was analyzed by Virology laboratory at Universidad Nacional Mayor de San Marcos (UNMSM)
Bluetongue virus	-80.426546	-3.635297	Sample was collected by Servicio Nacional de Sanidad Agraria del Perú (SENASA) and was analyzed by Virology laboratory at Universidad Nacional Mayor de San Marcos (UNMSM)
Bluetongue virus	-76.414763	-13.109883	Sample was collected by Servicio Nacional de Sanidad Agraria del Perú (SENASA) and was analyzed by Virology laboratory at Universidad Nacional Mayor de San Marcos (UNMSM)
Bluetongue virus	-75.47615059	-10.52002636	Sample was collected and analyzed by Virology laboratory at Universidad Nacional Mayor de San Marcos (UNMSM)
Bluetongue virus	-78.91057667	-6.16566833	Sample was collected and analyzed by Virology laboratory at Universidad Nacional Mayor de San Marcos (UNMSM)
Bluetongue virus	-72.72715416	-12.88909312	Sample was collected and analyzed by Virology laboratory at Universidad Nacional Mayor de San Marcos (UNMSM)
Bluetongue virus	-78.834005	-5.53074167	Sample was collected and analyzed by Virology laboratory at Universidad Nacional Mayor de San Marcos (UNMSM)
Bluetongue virus	-75.97893446	-9.22163807	Sample was collected and analyzed by Virology laboratory at Universidad Nacional Mayor de San Marcos (UNMSM)
Bluetongue virus	-78.695165	-5.65040833	Sample was collected and analyzed by Virology laboratory at Universidad Nacional Mayor de San Marcos (UNMSM)
Bluetongue virus	-76.819565	-6.765585	Sample was collected and analyzed by Virology laboratory at Universidad Nacional Mayor de San Marcos (UNMSM)
Bluetongue virus	-75.45971193	-9.64712841	Sample was collected and analyzed by Virology laboratory at Universidad Nacional Mayor de San Marcos (UNMSM)
Bluetongue virus	-69.48667333	-11.36169333	Sample was collected and analyzed by Virology laboratory at Universidad Nacional Mayor de San Marcos (UNMSM)
Bluetongue virus	-76.50476153	-7.2285323	Sample was collected and analyzed by Virology laboratory at Universidad Nacional Mayor de San Marcos (UNMSM)

Bluetongue virus	-69.28413354	-12.64252121	Sample was collected and analyzed by Virology laboratory at Universidad Nacional Mayor de San Marcos (UNMSM)
Bluetongue virus	-80.65275272	-3.67369242	Sample was collected and analyzed by Virology laboratory at Universidad Nacional Mayor de San Marcos (UNMSM)
Bluetongue virus	-80.24093767	-3.50815804	Sample was collected and analyzed by Virology laboratory at Universidad Nacional Mayor de San Marcos (UNMSM)
Culicoides insignis	-69.23952572	-12.59986631	Collected in the field by Universidad Nacional Mayor de San Marcos (UNMSM)
Culicoides insignis	-69.28413354	-12.64252121	Collected in the field by Universidad Nacional Mayor de San Marcos (UNMSM)
Culicoides insignis	-69.51029333	-11.46306167	Collected in the field by Universidad Nacional Mayor de San Marcos (UNMSM)
Culicoides insignis	-69.45027972	-11.3679079	Collected in the field by Universidad Nacional Mayor de San Marcos (UNMSM)
Culicoides insignis	-78.77555167	-5.614776667	Collected in the field by Universidad Nacional Mayor de San Marcos (UNMSM)
Culicoides insignis	-78.695165	-5.650408333	Collected in the field by Universidad Nacional Mayor de San Marcos (UNMSM)
Culicoides insignis	-76.49780092	-7.23780496	Collected in the field by Universidad Nacional Mayor de San Marcos (UNMSM)
Culicoides insignis	-76.793956	-6.813764	Collected in the field by Universidad Nacional Mayor de San Marcos (UNMSM)
Culicoides insignis	-76.813377	-6.76821	Collected in the field by Universidad Nacional Mayor de San Marcos (UNMSM)
Culicoides insignis	-75.42652324	-9.68119726	Collected in the field by Universidad Nacional Mayor de San Marcos (UNMSM)
Culicoides insignis	-75.47746545	-9.66754905	Collected in the field by Universidad Nacional Mayor de San Marcos (UNMSM)
Culicoides insignis	-75.50391295	-10.6267677	Collected in the field by Universidad Nacional Mayor de San Marcos (UNMSM)
Culicoides insignis	-75.48822408	-10.60754886	Collected in the field by Universidad Nacional Mayor de San Marcos (UNMSM)
Culicoides insignis	-75.4295399	-10.54386979	Collected in the field by Universidad Nacional Mayor de San Marcos (UNMSM)
Culicoides insignis	-75.40604477	-10.55367482	Collected in the field by Universidad Nacional Mayor de San Marcos (UNMSM)
Culicoides insignis	-74.5733	-8.8156	From literature: Dennis Navarro, M.; Miguel Rojas, M.; Jessica Jurado, P.; Alberto Manchego, S.; Mercy Ramírez, V.; Ana Castillo, E.; Hermelinda Rivera, G. Molecular Detection of Bluetongue Virus in *Culicoides insignis* and Sheep of Pucallapa, Peru. Revista de Investigaciones Veterinarias del Peru 2019, 30, 465–476, doi:10.15381/rivep.v30i1.15690.
Culicoides insignis	-78.426	-5.7364	From Specieslink: https://specieslink.net/search/download/20220916125420-0028877
Culicoides insignis	-78.649	-5.9592	From Specieslink: https://specieslink.net/search/download/20220916125420-0028877
Culicoides insignis	-73.2408	-3.7316	From Specieslink: https://specieslink.net/search/download/20220916125420-0028877
Culicoides insignis	-75.9162	-6.2639	From Specieslink: https://specieslink.net/search/download/20220916125420-0028877
Culicoides insignis	-69.553889	-12.703056	From Specieslink: https://specieslink.net/search/download/20220916125420-0028877
Culicoides insignis	-78.4442	-6.0894	From Specieslink: https://specieslink.net/search/download/20220916125420-0028877

Table S2. Set of bioclimatic variables used for construction the ENM for bluetongue virus.

Bioclimatic variables	Code	Set1	Set2	Set3	Set4
Mean annual air temperature	Bio1	X		X	
Mean diurnal air temperature range	Bio2	X		X	X
Isothermality	Bio3	X		X	X
Temperature seasonality	Bio4	X		X	X
Mean daily maximum air temperature air of the warmest month	Bio5	X	X		
Mean daily maximum air temperature air of the coldest month	Bio6	X	X		
Annual range of air temperature	Bio7	X	X		
Mean daily mean air temperature air of the wettest quarter	Bio8	X			X
Mean daily mean air temperature air of the driest quarter	Bio9	X			
Mean daily mean air temperature air of the warmest quarter	Bio10	X			
Mean daily mean air temperature air of the coldest quarter	Bio11	X			
Annual precipitation amount	Bio12	X	X	X	
Precipitation amount of the wettest month	Bio13	X	X		
Precipitation amount of the driest month	Bio14	X	X		
Precipitation seasonality	Bio15	X	X	X	X
Mean monthly precipitation amount of the wettest quarter	Bio16	X	X		
Mean monthly precipitation amount of the driest quarter	Bio17	X			
Mean monthly precipitation amount of the warmest quarter	Bio18	X	X		X
Mean monthly precipitation amount of the coldest quarter	Bio19	X		X	X

Table S3. Set of bioclimatic variables used for construction the ENM for *Culicoides insignis*.

Bioclimatic variables	Code	Set1	Set2	Set3	Set4	Set5
Mean annual air temperature	Bio1	X		X		X
Mean diurnal air temperature range	Bio2	X		X	X	
Isothermality	Bio3	X	X	X	X	
Temperature seasonality	Bio4	X		X	X	X
Mean daily maximum air temperature air of the warmest month	Bio5	X	X			X
Mean daily maximum air temperature air of the coldest month	Bio6	X				X
Annual range of air temperature	Bio7	X				X
Mean daily mean air temperature air of the wettest quarter	Bio8	X			X	
Mean daily mean air temperature air of the driest quarter	Bio9	X				
Mean daily mean air temperature air of the warmest quarter	Bio10	X				
Mean daily mean air temperature air of the coldest quarter	Bio11	X				
Annual precipitation amount	Bio12	X		X		X
Precipitation amount of the wettest month	Bio13	X	X		X	X
Precipitation amount of the driest month	Bio14	X				X
Precipitation seasonality	Bio15	X		X	X	
Mean monthly precipitation amount of the wettest quarter	Bio16	X				
Mean monthly precipitation amount of the driest quarter	Bio17	X				
Mean monthly precipitation amount of the warmest quarter	Bio18	X			X	
Mean monthly precipitation amount of the coldest quarter	Bio19	X	X	X	X	

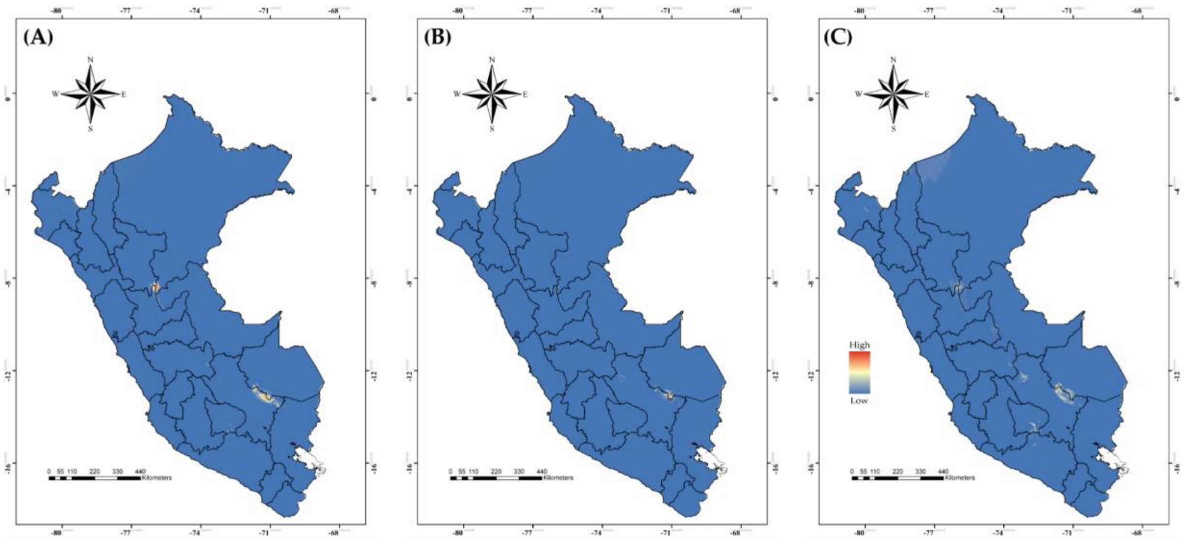

Figure S1. Model variability for bluetongue virus: median of variance coming from GCMs (A), SSPs (B) and replicates (C) in future climate scenerios.

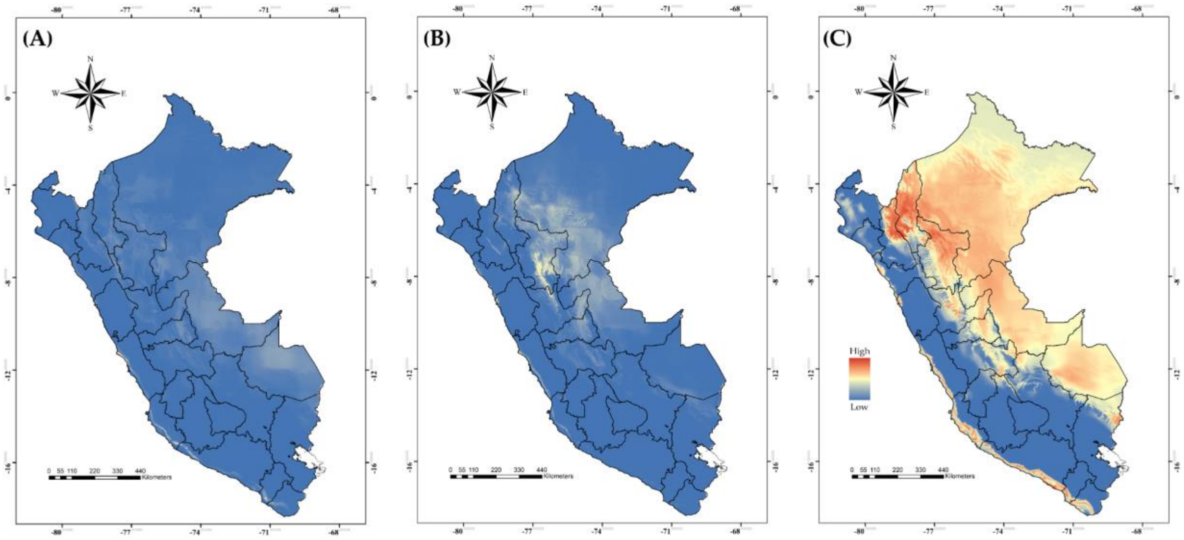

Figure S2. Model variability for *Culicoides insignis*: median of variance coming from GCMs (A), SSPs (B) and replicates (C) in future climate scenerios.

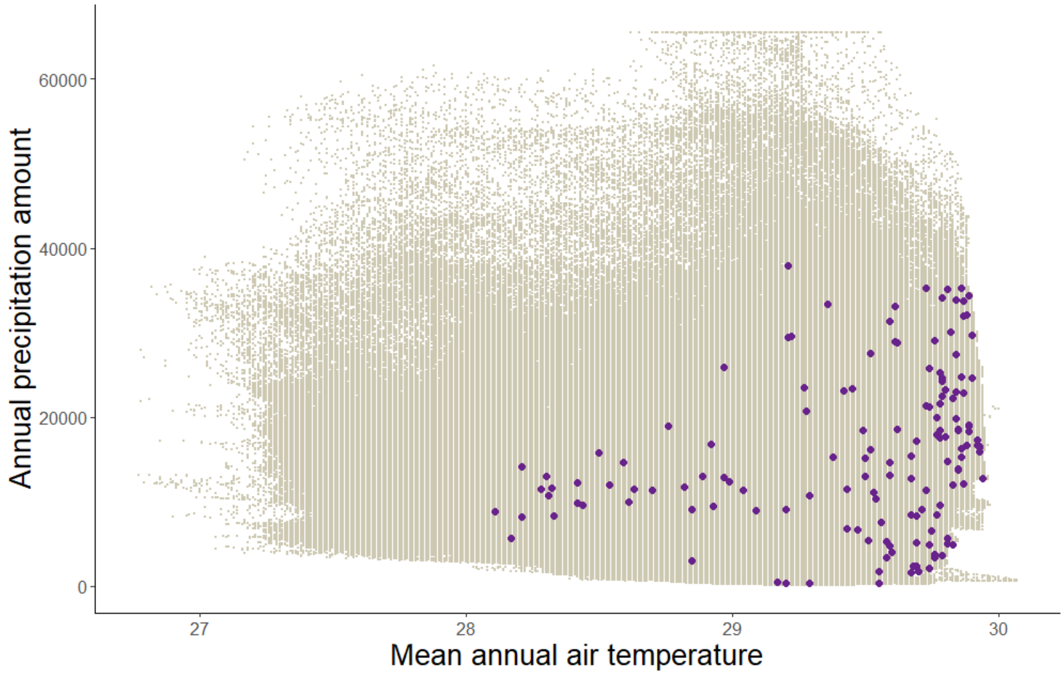

Figure S3. Scatterplot of bluetongue virus in an environmental space. Gray points are the projection area (Peru) while purple circles are used for occurrences in the ENM model.

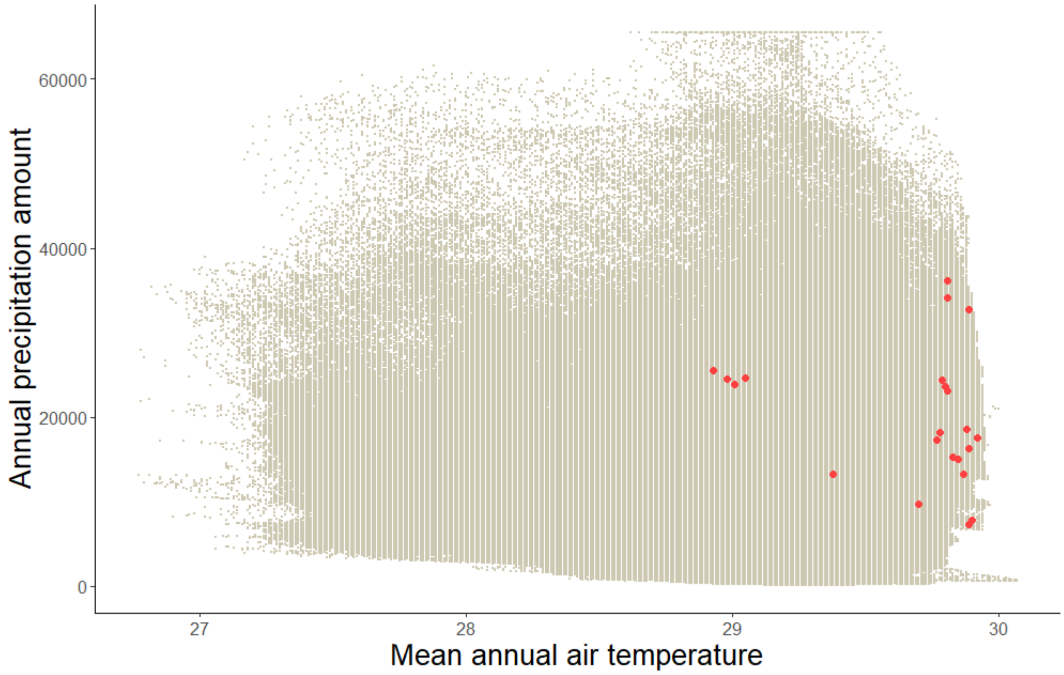

Figure S4. Scatterplot of *Culicoides insignis* in an environmental space. Gray points are the projection area (Peru) while red circles are used for occurrences in the ENM model
